# Beyond dead trees: integrating the scientific process in the Biodiversity Data Journal

**DOI:** 10.3897/BDJ.1.e995

**Published:** 2013-09-16

**Authors:** Vincent Smith, Teodor Georgiev, Pavel Stoev, Jordan Biserkov, Jeremy Miller, Laurence Livermore, Edward Baker, Daniel Mietchen, Thomas L.P. Couvreur, Gregory Mueller, Torsten Dikow, Kristofer M. Helgen, Jiři Frank, Donat Agosti, David Roberts, Lyubomir Penev

**Affiliations:** † The Natural History Museum, London, United Kingdom; ‡ Pensoft Publishers, Sofia, Bulgaria; § National Museum of Natural History, Sofia, Bulgaria; | Netherlands Centre for Biodiversity Naturalis, Leiden, Netherlands; ¶ Museum für Naturkunde – Leibniz-Institut für Evolutions- und Biodiversitätsforschung, Berlin, Germany; # Institut de Recherche pour le Développement (IRD), Montpellier, France; †† Chicago Botanic Garden, Glencoe, United States of America; ‡‡ National Museum of Natural History, Smithsonian Institution, Washington, DC, United States of America; §§ National Museum Prague, Prague, Czech Republic; || Plazi, Bern, Switzerland; ¶¶ Institute of Biodiversity and Ecosystem Research, Sofia, Bulgaria

## Introduction

Driven by changes to policies of governments and funding agencies, Open Access to content and data is quickly becoming the prevailing model in academic publishing. Open Access benefits scientists with greater dissemination and citation of their work, and provides society as a whole with access to the latest research. Open Access is, however, only one facet of scholarly communication. Core scientific statements or assertions are intertwined and hidden in the scholarly narratives, and the data underlying these statements are often obscured to the point that replication of results is impossible (Nature Editorial 2012). This is in part a result of the way scientific papers are written as narratives, rather than sources of data.

An often cited reason for the lack of published data is the absence of a reward mechanism for the individuals involved in creating and managing information (Smith 2009, Costello 2009, Vision 2010, McDade et al. 2011, Duke and Porter 2013). Preparing data for publication is a time consuming activity that few scholars will undertake without recognition from their peers. Data papers are a potential solution to this problem (Chavan and Penev 2011, Chavan and Penev 2013). They allow authors to publish data and receive reward through the traditional citation process. Coupling tools to rapidly and simply generate publications will incentivise this behaviour and create a culture of data curation and sharing within the biodiversity science community.

If we are going to incentivise the mass publication of data, we also need mechanisms to ensure quality. Traditional peer review is one of the bottlenecks in standard publication practice (Hauser and Fehr 2007, Fox and Petchey 2010). A common criticism of peer review is the lack of transparency and accountability on the part of the reviewers. To cope with the additional volume of papers created by data publication and to move to a more transparent system, we need to rethink peer review. We need both new methods of reviewing and new tools to automate as much of the review process as possible. This requires a new publishing platform, not just a new journal.

An abundance of small isolated datasets does not, however, allow us to address the fundamental problems within the biodiversity science community. These islands of data are only of value if connected and interlinked. The task of interlinking is performed by biodiversity data aggregators like the Global Biodiversity Information Facility (GBIF) and Encylopedia of Life (EOL) which form the backbone of data-driven biodiversity research. By automating the submission of data to these aggregators, we can increase their value to more than the sum of their parts, making small data big. A renewed appreciation of the value of small data will help to reduce the vast amount of research data that exists only on laptops and memory sticks - data that is often lost when people change roles or retire.

Works of potentially very limited length can hold intrinsic value to the community, but are almost impossible to publish in traditional journals chasing impact factors. Examples include single species descriptions, local checklists and software descriptions, or ecological surveys and plot data. An infrastructure that allows datasets of any size to be important means we can publish them at any time. There is no need to wait for datasets to reach a critical mass suitable for publication in a traditional journal.

Today, we are pleased to announce the official release of the first series of papers published in Biodiversity Data Journal (BDJ). After years of hard work in analyzing, planning and programming the Pensoft Writing Tool (PWT), we now have a publishing platform that addresses the key concerns raised above. This provides the first workflow to support the full life cycle of a manuscript - from writing through submission, community peer-review, publication and dissemination, all within a single online collaborative environment.

### Shortening distance between “data” and “narrative” publishing

Most journals nowadays clearly separate data from narrative (text). Moreover, data publishing through data centres and repositories has almost become a separate sector within the scholarly publishing landscape. BDJ is not a conventional journal, nor is it a conventional “data journal”. It aims to integrate data and text in a single publication by converting several kinds of biodiversity data (e.g., species occurrences, checklists, or data tables) into the text for human-readable use, while simultaneously making data units from the same article harvestable and downloadable. The text itself is marked up and presented in a highly structured and machine readable form.

BDJ aims to integrate small data into the text whenever possible. Supplementary data files that underpin graphs, hypotheses and results can also be uploaded on the journal’s website and published with the article.

Nonetheless, this is usually not possible for large or complex data, for which we recommend deposition in an established open international repository (for details, see Penev et al. 2011):

- Large primary biodiversity data sets (e.g., institutional collections of species-occurrence records) should be published with the GBIF Integrated Publishing Toolkit (IPT); small data sets of this kind are imported into the article text through an Excel template, available in PWT.
- Genomic data should be deposited with INSDC (GenBank/EMBL/DDBJ), either directly or via a partnering repository, e.g. Barcode of Life Data Systems (BOLD). Transcriptomics data should be deposited in Gene Expression Omnibus (GEO) or ArrayExpress.
- Phylogenetic data should be deposited at TreeBASE, either directly or through the Dryad Data Repository.
- Biodiversity-related geoscience and environmental data should be deposited in PANGAEA.
- Morphological images other than those presented in the article should be deposited at Morphbank. Images of a specific kind should be deposited in appropriate repositories if these exist (e.g., Morphosource for MicroCT data).
- Videos should be uploaded to video sharing sites like YouTube, Vimeo or SciVee and linked back to the article text. Similarly, audio files should go to platforms like FreeSound or SoundCloud, and presentations to Slideshare. In addition, multimedia files can also be uploaded as supplementary files on the journal’s website. 3D and other interactive models can be embedded in the article’s HTML and PDF.
- Any other large data sets (e.g., ecological observations, environmental data, morphological and other data types) should be deposited in the Dryad Data Repository, either prior to or upon acceptance of the manuscript. Other specialised data repositories can be used if these offer unique identifiers and long-term preservation.

All external data used in a BDJ paper must be cited in the reference list, and links to these data (as deposited in external repositories) must be included in a separate data resources section of the article.

All datasets, images or multimedia are freely downloadable from the text under the Open Data Commons Attribution License or a Creative Commons CC-Zero waiver / Public Domain Dedication. The article text is available under a Creative Commons (CC-BY) 3.0 license. Primary biodiversity data within an article can be exported in Darwin Core Archive format, which makes them interoperable with biodiversity tools based on the Darwin Core standard.

By facilitating open access to the data that underlie every publication, BDJ is setting a new standard in transparency and repeatability in biodiversity science. Perpetual and universal access to primary data stimulates scientific progress by helping authors build upon existing datasets. BDJ’s commitment to supporting automated data aggregation and interlinking is happening alongside multiple advances in biodiversity informatics infrastructure that herald the dawning of an era of collaborative, big-data biodiversity science (Page 2008, Patterson et al. 2010, Thessen and Patterson 2011, Parr et al. 2012).

### Authoring, peer-review and publication in one place, for the first time

**Figure 1.**
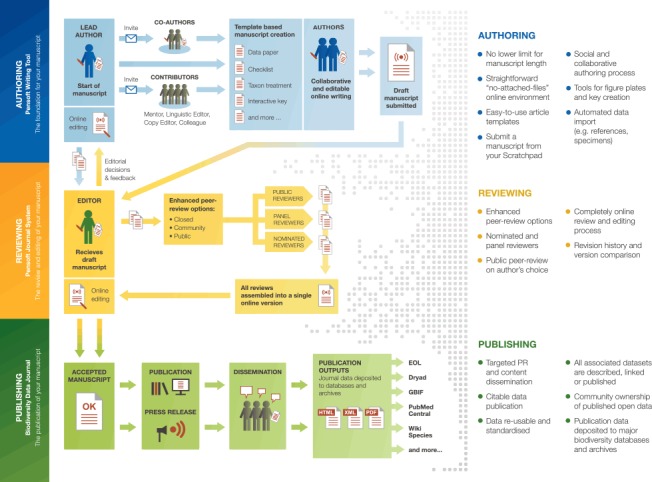
Three-step editorial workflow in the Biodiversity Data Journal: manuscript authoring, peer-review and publication.

The online, collaborative, article-authoring platform (Pensoft Writing Tool, PWT) is the principal way to write and submit a manuscript to BDJ. It provides a set of pre-defined, but flexible article templates (Fig. 1). Authors may work collaboratively on a manuscript and invite external contributors, such as mentors, potential reviewers, linguistic and copy editors. Colleagues may read and comment on the text before submission. Images are arranged into plates through a plate builder. This allows component images to be individually labeled, viewed, enlarged, linked to content, embedded, downloaded or otherwise used and reused.

A special feature of PWT is that the authors can see at any time an editable preview of their manuscript in a format that is very close to the final published version. On completion of the manuscript, it can be submitted to the journal with a simple click of a button that will initiate the review process. The tool also allows automated import of manuscripts from data management platforms such as Scratchpads. Several tools in PWT facilitate import of data, references, images and other data.

A major advantage of the PWT is that it handles much of the semantic enhancement of a manuscript automatically during validation, eliminating the need for the authors or editors to manually markup portions of text. Examples of this include taxonomic names and georeferenced localities. The validation tool checks for compliance with the relevant biological code, for example checking that a holotype designation has been made for a new species description and that a new genus has a designated type species. In the near future, the PWT will also automatically register nomenclatural acts in the appropriate registry (International Plant Names Index, Index Fungorum, MycoBank or ZooBank).

The technology used by the PWT largely eliminates the conventional layout stage, just as the validation tool saves work for the copyeditors. Our goal is to greatly reduce the publication costs for all. This is particularly important because many authors working within biodiversity science are not backed by large institutions who can cover large page charges.

A novel *community-based* peer review of the manuscripts submitted to BDJ provides the opportunity for many specialists in the field to review a manuscript. The purpose of community peer review is to distribute effort, increase speed and transparency, engage the broader community of experts, and enhance the quality of the science we publish.

There are three groups of reviewers that may participate in the community peer review process: *nominated*, *panel*, and *public reviewers*. Nominated reviewers are expected to agree to provide a formal review by a deadline, and in this sense, they operate in the same way as conventional referees in most other journals. Panel reviewers are also invited to evaluate the manuscript, but without the formal acceptance of the deadline. They can submit their review, if they wish, at any time before the editorial process is finalised. Both nominated and panel reviewers can propose changes and corrections, make comments in the manuscript online and submit a concise reviewers’ evaluation form. Reviewers may opt to be anonymous but we encourage them to disclose their names. In the near future, authors will be able to opt for an entirely public peer-review process. Finally, comments can be posted after publication, so as to extend the review process even further and to enrich it with new insights, corrections or follow-up work.

**Figure 2.**
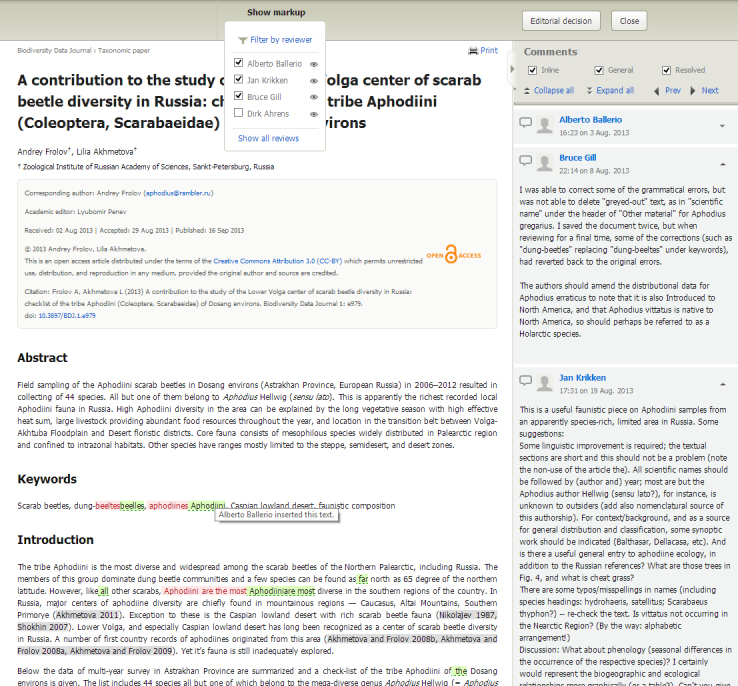
Consolidated review version. The editor sees all reviewer's corrections and comments in one place and can filter them out. The editor can also insert his/her own corrections and comments before submitting the editorial evaluation.

The editor’s work is reduced by a tool that collates reviewers’ comments and corrections into a single document. Upon receipt of this consolidated review and editorial evaluation (Fig. 2), the authors may accept or reject the proposed corrections, reply to comments of the reviewers and edit their manuscript in the same single online document for one-click resubmission.

Accepted articles are published in semantically enhanced HTML, PDF and XML versions, compliant with the TaxPub schema, an extension of the NLM/NCBI Journal Article Tag Suite (JATS) used by the PubMedCentral archive (Catapano 2010).

### Delivering appropriate content to different users

In the Internet era, dissemination of published information is at least as important as the act of publishing. The highly structured text, domain-specific markup and underlying data can be used not only for effective reading but also to provide users direct access to the precise data they need (Penev et al. 2010). For example, an essential part of systematics publications are taxon treatments. In the BDJ these are automatically extracted from the text and submitted for display and further re-use in the Encyclopedia of Life, the Plazi Treatment Repository and the wiki-based repository Species-ID.

Literature references are exported to the community-owned Bibliography of Life (based on the RefBank database and the ReFinder bibliographic search tool) as well as to several other bibliographic databases. This allows for their further re-use and import into new publications, saving authors a great deal of time locating historical literature.

Images are exported to Encyclopedia of Life, which increases their visibility and re-use.

### Are the “small” data really small?

Costello et al. (2013) recently called for the publication, citation and peer review of biodiversity data. The platform we have built addresses all of these concerns in one easy-to-use and integrated solution that also increases the speed and transparency of the publication process. By automating as much as possible, we will significantly reduce the costs of Open Access, maintain rigorous standards and make a major step toward integrating biodiversity data. The BDJ is not just a new journal. It is a revolutionary model in academic publication practice that will make a major step toward realising the full potential of biodiversity data.
